# Ruptured A3 segment aneurysm in a patient with neurofibromatosis type 1: a case report and literature review

**DOI:** 10.3389/fsurg.2026.1825953

**Published:** 2026-06-25

**Authors:** Fang Xue, Fei Xie, Jianqiang Hao, Sen He

**Affiliations:** Department of Neurosurgery, West China Hospital of Sichuan University-Ziyang Hospital & Ziyang Central Hospital, Ziyang, Sichuan, China

**Keywords:** anterior cerebral artery, endovascular coil embolization, intracranial aneurysm, long-term follow-up, moyamoya syndrome, neurofibromatosis type 1, vasculopathy

## Abstract

**Background:**

Neurofibromatosis type 1 (NF1) is a genetic disorder occasionally complicated by vasculopathy, but the concurrence of NF1, a ruptured distal anterior cerebral artery (ACA) aneurysm, and moyamoya syndrome is exceedingly rare. Literature review revealed that fewer than 20 such cases have been reported in English publications to date. The management of such complex, multifocal cerebrovascular pathology—including the choice of therapy, the risk of procedural complications, and the need for lifelong surveillance—poses significant challenges.

**Case description:**

A 44-year-old man with clinical features of NF1 presented with sudden headache, dizziness, and transient syncope. Imaging revealed a ruptured saccular aneurysm arising from the right A3 segment of the ACA, accompanied by intraventricular hemorrhage and contralateral moyamoya-like vasculopathy. Given the aneurysm's deep pericalosal location, small size, and the patient's multiple scalp neurofibromas, endovascular coil embolization was selected over microsurgical clipping. The procedure was successfully performed via a transfemoral approach, achieving complete aneurysm occlusion with preservation of the parent artery. The patient recovered well neurologically. Remarkably, digital subtraction angiography at more than five years post-embolization confirmed durable occlusion of the treated aneurysm without recurrence. However, it also demonstrated progression of the moyamoya syndrome and revealed a new, untreated aneurysm at the right ophthalmic segment.

**Conclusion:**

This case illustrates that endovascular coil embolization can be a safe and effective first-line treatment for ruptured distal ACA aneurysms in the complex setting of NF1 vasculopathy, providing long-term protection from rebleeding. The long-term follow-up uniquely highlights that NF1-associated cerebrovascular disease is progressive and multifocal; successful treatment of an index ruptured aneurysm does not eliminate the risk of new lesions elsewhere. Therefore, indefinite vascular surveillance is warranted in these patients. This report underscores the importance of a tailored, multidisciplinary approach and lifelong monitoring in managing NF1-related cerebrovascular complications.

## 1 Introduction

Neurofibromatosis type 1 (NF1) is a common autosomal dominant genetic disorder with an estimated prevalence of approximately 1 in 2,500–3,000 individuals worldwide (1–3). Its typical clinical manifestations include café-au-lait macules, axillary or inguinal freckling, cutaneous neurofibromas, Lisch nodules, optic pathway glioma, and a spectrum of skeletal abnormalities (4–6). Vascular involvement is far less common, with reported frequencies ranging from 0.4% to 6.4%, but it is clinically important because the resulting lesions may include aneurysms, arterial stenosis or occlusion, moyamoya syndrome, and arteriovenous fistulas (7–11). Compared with peripheral aneurysms, intracranial aneurysms in NF1 appear to be particularly rare (12).

Distal anterior cerebral artery aneurysms involving the A3 segment are themselves uncommon (13). This segment courses around the genu of the corpus callosum and transitions between the pericallosal and callosomarginal arterial territories, making surgical exposure technically demanding because of the deep location and adjacent branching pattern (14). Once rupture occurs, the risk of severe neurological disability or death is substantial (15). When NF1, distal anterior cerebral artery aneurysm, and moyamoya-like vasculopathy coexist in the same patient, diagnosis and treatment become even more challenging, and reports remain limited.

Here, we present a patient with NF1 who developed a ruptured right A3 segment aneurysm and underwent successful endovascular coil embolization, with angiographic follow-up extending to April 30, 2024. The case is rewritten in a standard English scientific format and discussed in the context of the available literature.

Neurofibromatosis type 1 is known to predispose patients to vasculopathy through several mechanisms, including abnormal proliferation of vascular smooth muscle cells, dysregulation of the Ras signaling pathway, and secondary endothelial dysfunction. These pathological changes contribute to the development of intracranial aneurysms and moyamoya-like vascular changes in affected individuals.

## 2 Case presentation

### 2.1 Initial presentation and diagnostic assessment

A 44-year-old man was admitted to our hospital on March 14, 2019, because of sudden headache and dizziness lasting for more than 1 h. He experienced one episode of transient syncope that lasted approximately 5–6 min and recovered spontaneously, after which nausea, slowed memory, and delayed thinking became apparent. He had no documented history of hypertension, diabetes mellitus, or infectious disease. His family history was unremarkable, and no relatives were known to have NF1 or intracranial aneurysm.

**Figure 1:**
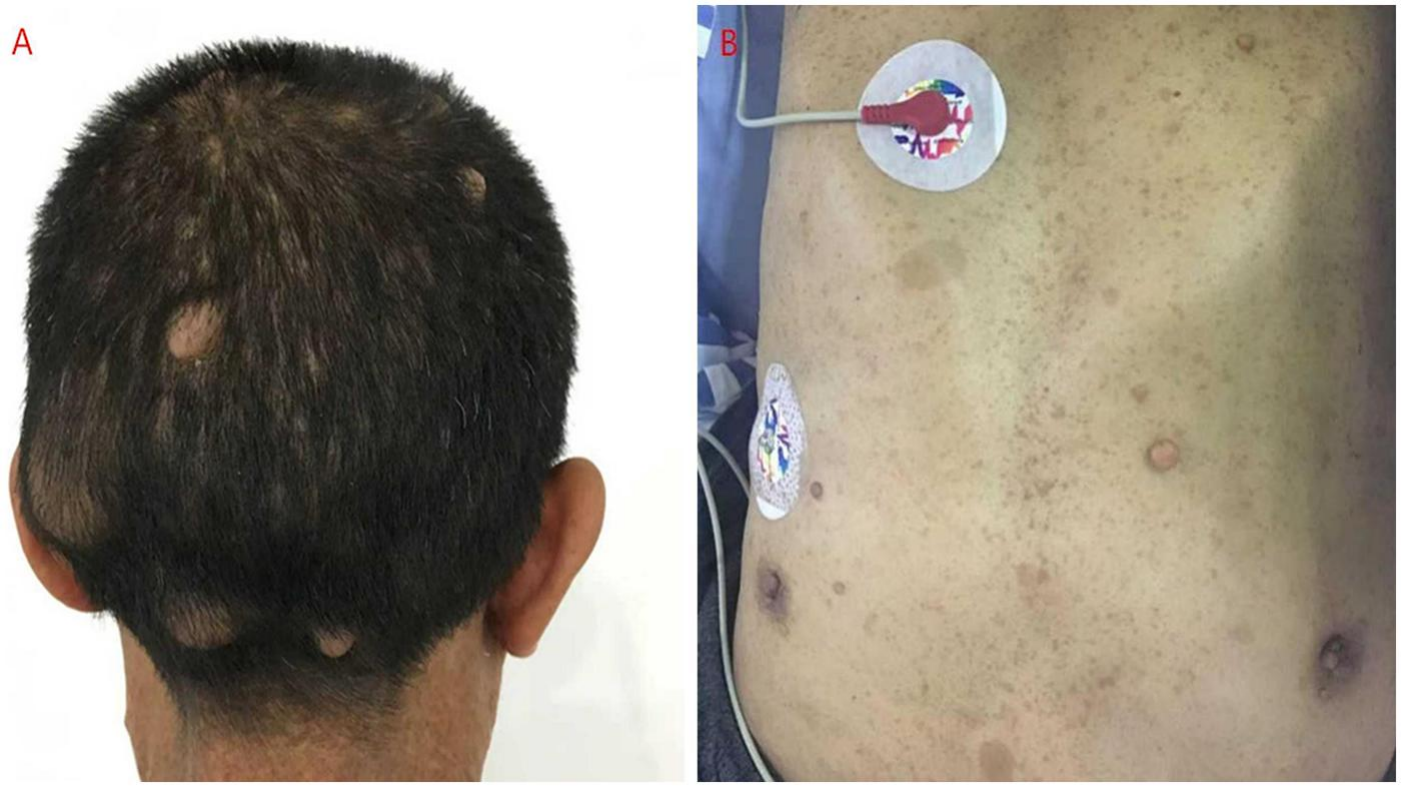
Clinical manifestations of neurofibromatosis type 1. **(A)** Multiple neurofibromas over the occipital region of the head and neck. **(B)** Scattered café-au-lait macules and small cutaneous tags.

On examination, the patient was drowsy but answered questions appropriately and could follow simple commands, with a Glasgow Coma Scale (GCS) score of 14/15 (E3V5M6). The pupils were equal and reactive to light, with an estimated diameter of 3 mm bilaterally. Facial symmetry was preserved, the tongue was midline, and muscle strength and tone were normal in all four extremities. Based on the patient's clinical status and GCS score, the initial clinical grade was classified as WFNS Grade II, and Hunt and Hess Grade II. Multiple soft masses were palpable over the head, neck, and especially the occipital region. The overlying skin was coarse and hyperpigmented, with scattered café-au-lait macules and small cutaneous tags (Figure 1). In the clinical context of multiple neurofibromas and typical pigmentary lesions, the diagnosis was consistent with NF1 according to the revised diagnostic criteria (16).

**Figure 2:**
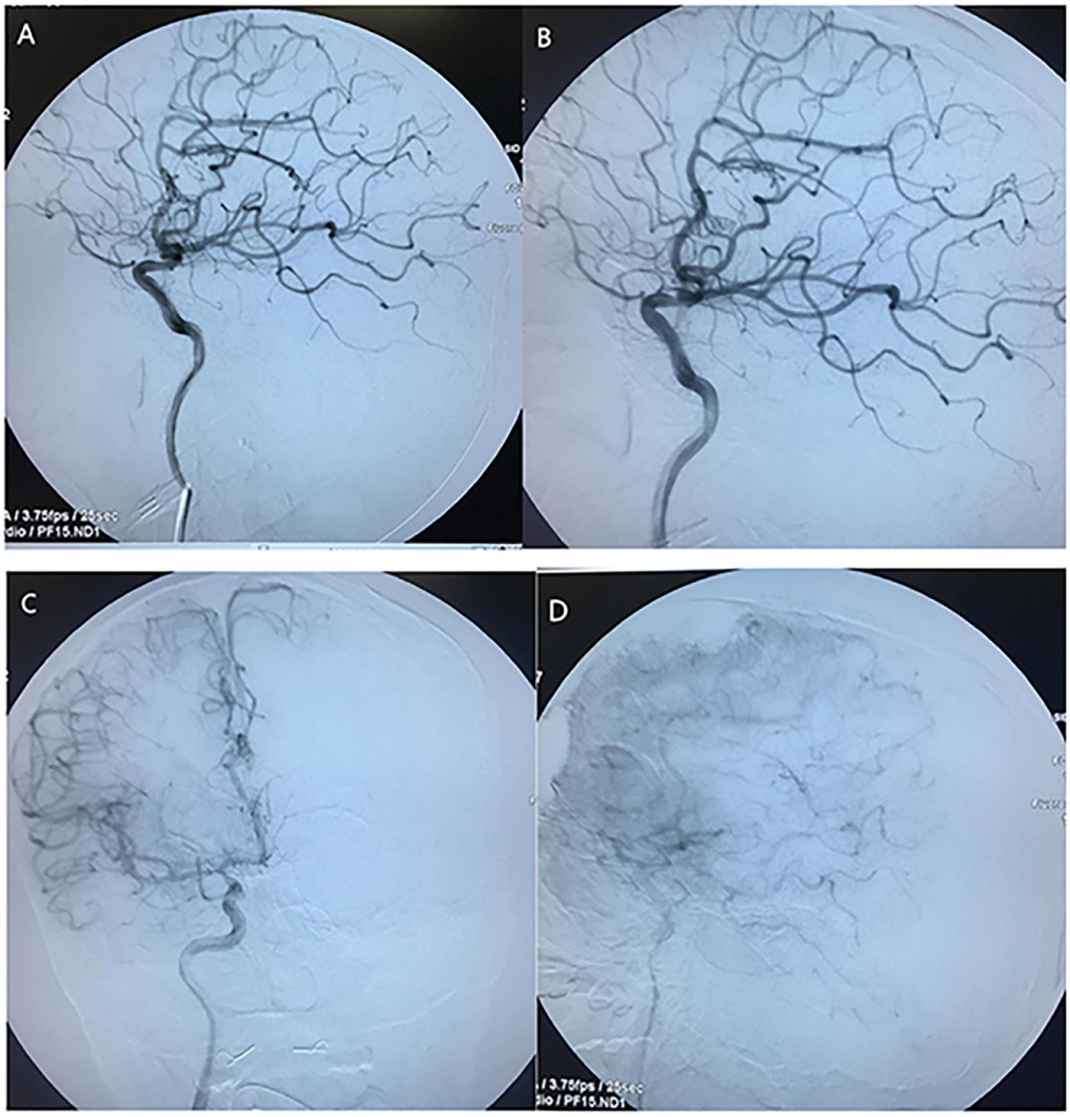
Preoperative imaging findings. **(A)** Cranial CT showing a hyperdense lesion in the corpus callosum region with intraventricular hemorrhage. **(B–D)** Cranial CTA demonstrating an aneurysm in the A3 segment of the anterior cerebral artery.

Initial cranial computed tomography (CT) combined with computed tomography angiography (CTA) showed a nodular lesion in the region of the corpus callosum, approximately 0.3 cm in diameter, with enhancement similar to that of adjacent arteries and close relation to a branch of the anterior cerebral artery. The lesion was considered most consistent with a ruptured branch aneurysm and was accompanied by intraventricular hemorrhage. Additional findings included a left temporal arachnoid cyst with adjacent calvarial resorption, focal atrophy of the left parietal gyrus with widening of the adjacent subarachnoid space, and diffuse attenuation of the left internal carotid artery and its major branches compared with the right side (Figure 2). The admission CT showed subarachnoid hemorrhage with intraventricular extension, corresponding to Fisher Grade 4.

### 2.2 Therapeutic intervention

On March 17, 2019, the patient underwent whole-brain angiography followed by coil embolization of the right anterior cerebral artery aneurysm. Intraoperative angiography demonstrated a saccular aneurysm arising from the right A3 segment, with an estimated size of approximately 2 mm × 3 mm × 2 mm. The terminal branches of the left internal carotid artery were attenuated, a finding consistent with moyamoya syndrome.

**Figure 3:**
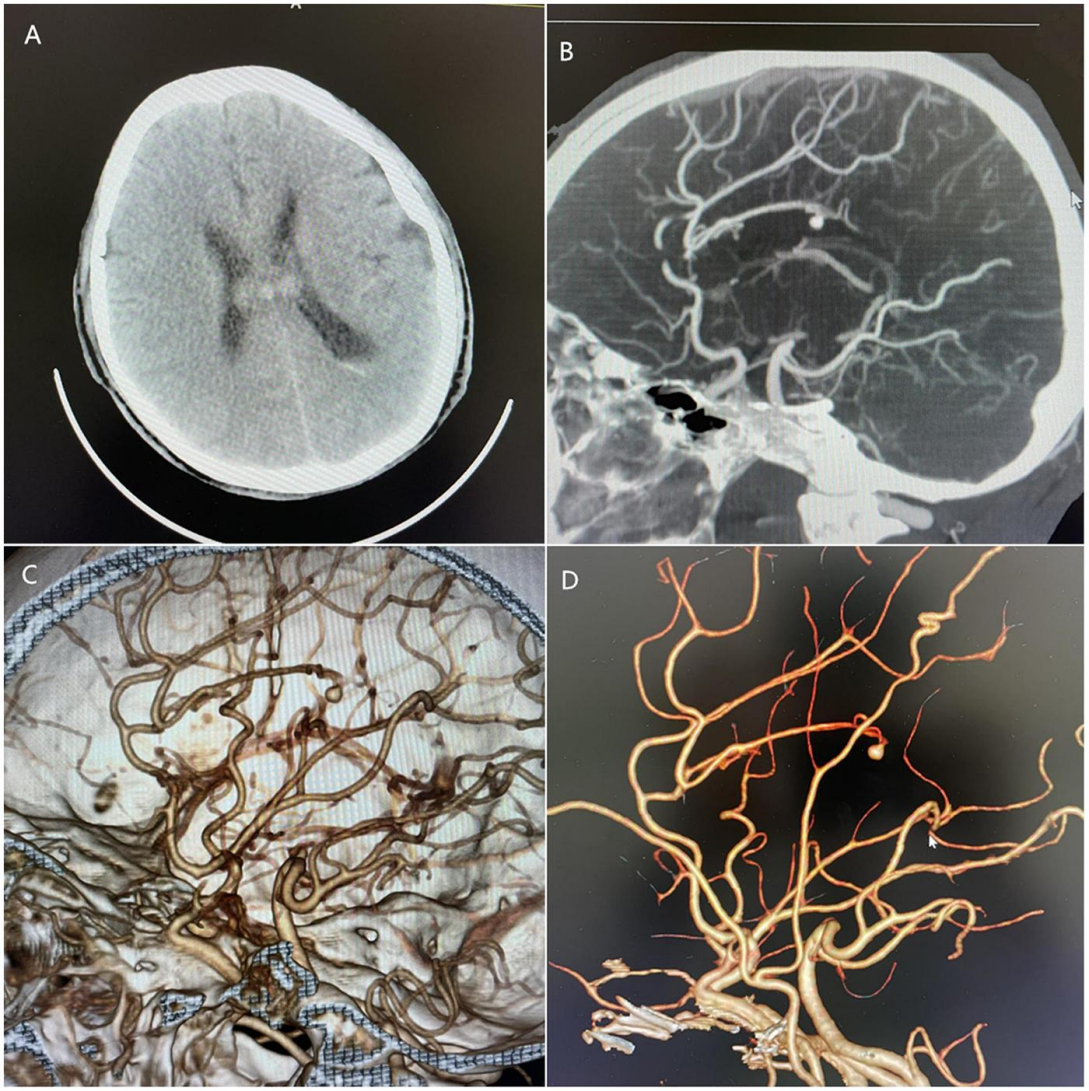
Intraoperative angiography and embolization. **(A)** Diagnostic digital subtraction angiography. **(B–D)** Coil embolization of the ruptured A3 aneurysm.

Using a standard Seldinger technique, the right femoral artery was punctured and a 6F arterial sheath was introduced. After systemic heparinization, a guiding catheter was advanced into the right internal carotid artery. Once the aneurysm had been localized angiographically, an appropriate working projection was selected. A shaped microcatheter was navigated into the aneurysm sac under microwire guidance, after which detachable coils were deployed until satisfactory packing was achieved. Final angiography demonstrated disappearance of aneurysm opacification, no contrast extravasation, and preserved patency of the parent artery (Figure 3).

Due to the distal location of the aneurysm at the A3 segment, the vascular course was long and tortuous with a small-caliber parent artery and a narrow aneurysm neck. These factors made it technically challenging to advance and stabilize the microcatheter within the aneurysm sac. In addition, the small size of the aneurysm raised potential risks of intraprocedural rupture during coiling, as well as coil herniation into the parent artery, which might cause distal vessel occlusion and subsequent cerebral infarction. To reduce these risks, we carefully selected suitable working projections and slowly filled the soft spring coil under continuous radiation monitoring. Post-procedural angiography was conducted for final verification. No perioperative complications were noted, including perforator infarction and distal embolization.

The postoperative diagnoses were as follows: (1) ruptured hemorrhagic aneurysm of the right A3 segment; (2) NF1 complicated by moyamoya syndrome; (3) intraventricular hemorrhage; and (4) left temporal arachnoid cyst.

### 2.3 Postoperative course and follow-up

After the procedure, the patient received symptomatic and supportive treatment, including prevention of cerebral vasospasm, volume expansion, antiepileptic treatment, fluid replacement, and correction of electrolyte imbalance. His condition improved gradually. He was discharged in good condition on April 10, 2019.

Between 2019 and 2024, the patient was followed up annually via telephone or outpatient visits. He remained clinically stable with no new neurological symptoms or adverse events. However, due to financial constraints, the patient declined all recommended vascular imaging examinations during this period.

**Figure 4:**
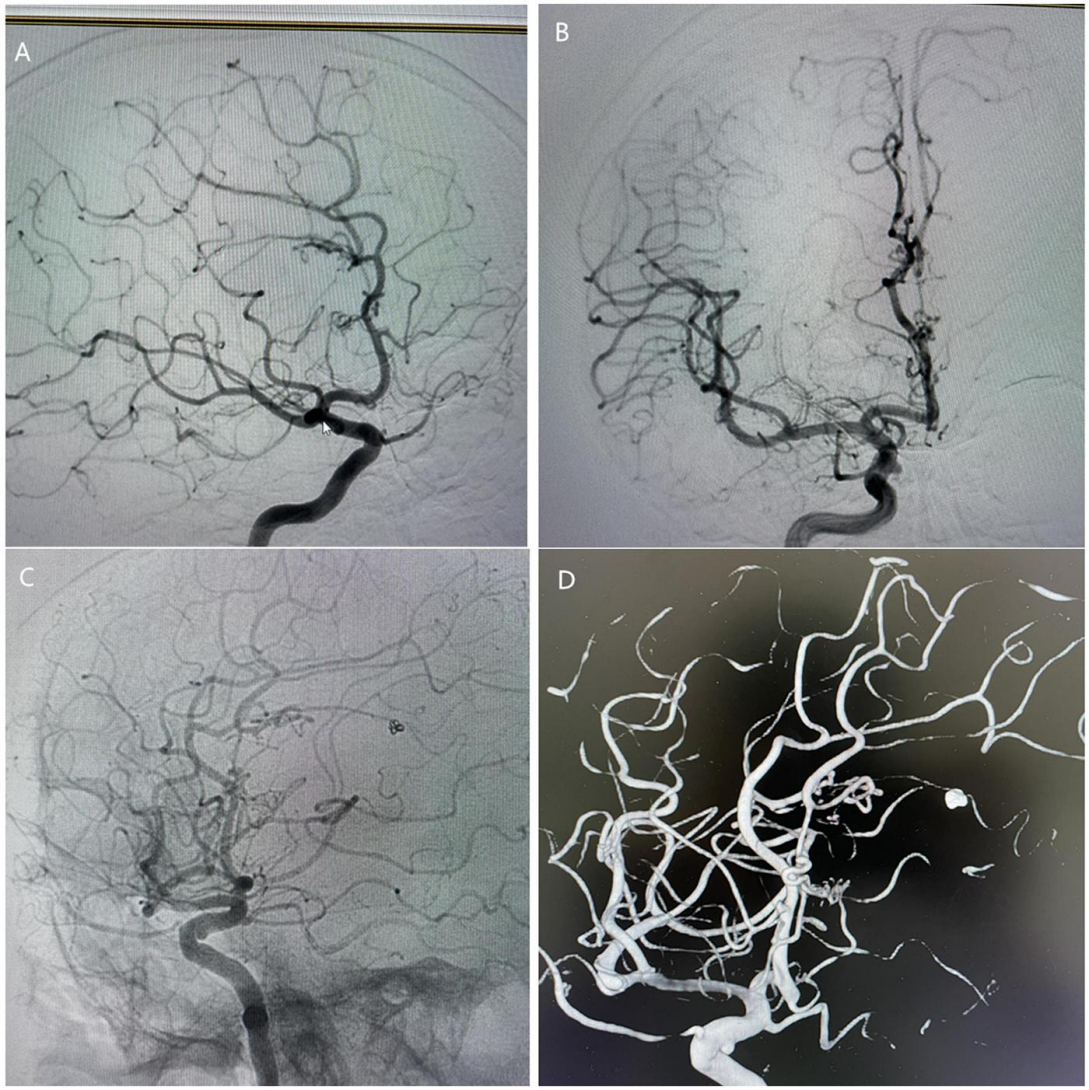
Follow-up digital subtraction angiography obtained on April 30, 2024, showing durable occlusion of the treated A3 aneurysm, preservation of the parent artery, and persistent multifocal cerebrovascular abnormalities. **(A)** DSA anteroposterior view; **(B)** DSA lateral view; **(C)** DSA oblique view; **(D)** DSA 3D reconstruction.

At follow-up on April 30, 2024, the patient returned for re-evaluation. He was fully alert and answered questions appropriately. Both pupils measured approximately 3 mm and were reactive to direct and consensual light. Muscle strength and tone were normal in all extremities. The Mini-Mental State Examination score was 30, and the Montreal Cognitive Assessment score was also 30. Digital subtraction angiography demonstrated stable post-embolization changes in the right A3 aneurysm, a patent parent artery, and no residual filling or recurrence. However, an additional aneurysm was identified in the right ophthalmic segment of the internal carotid artery, with a neck of approximately 3.22 mm and sac dimensions of approximately 3.25 mm × 2.43 mm × 1.32 mm. The left terminal internal carotid artery was occluded, with angiographic findings consistent with moyamoya syndrome: the anterior cerebral artery territory was supplied by compensation from the ipsilateral ophthalmic artery and contralateral anterior cerebral artery, whereas the middle cerebral artery territory received collateral flow from the bilateral vertebral arteries via the posterior cerebral arteries (Figure 4). Treatment for the ophthalmic-segment aneurysm was recommended because of the high-risk vascular background, but the patient declined surgery for financial reasons. He improved with conservative management and was discharged; no further in-person follow-up has been documented to date. The timeline of the main clinical events of the patient is shown in Table 1.

**Table 1:** Chronological timeline of major clinical events.

Date	Clinical event
March 14, 2019	Admission because of abrupt headache, dizziness, transient syncope, and nausea. CT/CTA suggested ruptured distal anterior cerebral artery aneurysm with intraventricular hemorrhage and asymmetry of the internal carotid arteries.
March 17, 2019	Diagnostic cerebral angiography confirmed a right A3 aneurysm and moyamoya-like changes; endovascular coil embolization was performed.
April 10, 2019	The patient was discharged after clinical improvement.
April 21, 2020	The patient was followed up by phone and refused to undergo cerebrovascular examination due to financial reasons.
May 12, 2021	The patient was followed up by phone and refused to undergo cerebrovascular examination due to financial reasons.
March 15, 2022	The patient was followed up in the outpatient department, but refused to undergo cerebrovascular examination due to financial reasons.
May 10, 2023	The patient was followed up by phone and refused to undergo cerebrovascular examination due to financial reasons.
April 30, 2024	Follow-up digital subtraction angiography demonstrated durable occlusion of the treated A3 aneurysm without recurrence, preserved parent artery patency, a right ophthalmic-segment aneurysm, and persistent moyamoya syndrome.

## 3 Discussion

This case highlights the rare coexistence of NF1, a ruptured distal anterior cerebral artery aneurysm, and moyamoya syndrome. NF1-associated vasculopathy is well recognized but distinctly uncommon in daily practice (17). When cerebrovascular lesions do occur, they may involve aneurysm formation, progressive arterial stenosis or occlusion, and collateral remodeling. The simultaneous presence of an A3 aneurysm and moyamoya-like changes in our patient supports the concept that NF1-related vasculopathy can be multifocal rather than confined to a single vascular segment.

From a mechanistic standpoint, NF1 results from pathogenic alterations of the *NF1* gene on chromosome 17q11.2 (18). The encoded protein, neurofibromin, negatively regulates the Ras/MAPK pathway by accelerating the conversion of Ras-GTP to Ras-GDP. Loss of neurofibromin function leads to excessive Ras signaling, dysregulated cellular proliferation, and disordered vascular homeostasis (19–21). Previous studies have suggested that these abnormalities may affect vascular smooth muscle cells, elastic fiber assembly, and structural integrity of the vascular media, thereby predisposing patients to aneurysm formation, stenosis, occlusion, arteriovenous fistulas, and moyamoya-like vascular remodeling (9, 22–26). However, the patient refused to undergo genetic testing.

In addition to the intrinsic medial weakness caused by NF1, the coexistence of moyamoya syndrome in this patient further amplifies vascular fragility and rupture risk. Moyamoya vasculopathy is characterized by progressive stenosis or occlusion of the terminal internal carotid arteries, which induces the formation of fragile collateral vessels to compensate for cerebral hypoperfusion. Histopathological studies have shown that these leptomeningeal and perforating collaterals often exhibit attenuated walls, fragmented elastic laminae, and deficient smooth muscle layers, making them structurally vulnerable to hemodynamic stress (23, 26).

In this case, the ruptured aneurysm involved the A3 segment of the anterior cerebral artery—a territory that frequently serves as part of the collateral network in moyamoya syndrome. We hypothesize that the combination of ① systemic connective tissue dysplasia due to NF1, ② chronic hemodynamic burden imposed by moyamoya-induced stenosis, and ③ the inherent structural fragility of the distal and collateralized ACA branches created a high-risk environment for saccular aneurysm formation and subsequent rupture. This mechanistic framework helps explain why the aneurysm occurred in a distal, hemodynamically challenged vessel rather than in typical anterior circulation locations.

To place our findings within the context of published evidence, we reviewed the literature cited in this report. A systematic analysis by Bargiela et al. (27), referenced herein as part of the broader NF1 vasculopathy discussion, summarized 66 patients with neurofibromatosis-associated aneurysms from 59 articles. Crucially, this synthesis revealed that intracranial localization accounted for only a minority of cases (∼4.5%), with the vast majority affecting aortic, renal, and visceral arteries. Unlike the predominant involvement of proximal anterior circulation segments (e.g., internal carotid artery ophthalmic segment, middle cerebral artery bifurcation) reported in prior NF1 intracranial aneurysm series (12, 27), our case uniquely presented with a ruptured aneurysm in the distal A3 segment—a location noted to be “itself uncommon” (13) and scarcely documented even in NF1 patients with moyamoya syndrome (8). Among the intracranial cohort, anterior circulation aneurysms predominated. Regarding treatment modalities, endovascular coil embolization was the most frequently reported technique, utilized in approximately 83% of intervened cases, while microsurgical clipping was reserved for select anatomies. In contrast to the general preference for microsurgical clipping in accessible lesions (14), our case demonstrates the superiority of endovascular embolization for a small (≈2mm × 3mm × 2 mm), deep-seated A3 aneurysm: the narrow operative corridor around the corpus callosum constrained surgical exposure, multiple scalp neurofibromas raised craniotomy-related wound risks, and the absence of hydrocephalus/large hematoma eliminated the need for open decompression. Although major complication rates were reported around 15% in that series, robust data specifically comparing long-term recurrence rates between endovascular and surgical approaches for NF1-related intracranial aneurysms remain scarce. This literature underscores that while NF1 vasculopathy is well-documented, symptomatic intracranial aneurysms represent an exceptionally rare phenotypic expression, often occurring in the setting of concurrent moyamoya syndrome—a pattern consistent with the present case (8, 23).

The therapeutic priority in this case was immediate management of the ruptured aneurysm, because aneurysmal subarachnoid or intraventricular hemorrhage carries a high risk of rebleeding, disability, and death (28, 29). Given the underlying vascular fragility associated with both NF1 and moyamoya syndrome, endovascular obliteration of the ruptured lesion was considered essential to prevent catastrophic rebleeding from another fragile collateral vessel. In principle, both microsurgical clipping and endovascular embolization can be considered for distal anterior cerebral artery aneurysms (14, 30). However, several factors favored an endovascular strategy here. First, the aneurysm was small and located deeply around the corpus callosum, where microsurgical exposure is constrained by the narrow operative corridor and adjacent arterial branches. Second, the patient had multiple scalp and occipital neurofibromas, raising concern that craniotomy might increase the risk of wound-healing problems, skin ischemia, or infection. Third, no hydrocephalus, significant intracranial hematoma, or major mass effect was evident on the admission CT, reducing the need for open decompressive surgery. Under these circumstances, coil embolization offered a minimally invasive means of securing the aneurysm while limiting procedure-related trauma.

The long-term angiographic outcome is clinically informative. More than 5 years after treatment, the previously ruptured A3 aneurysm remained completely occluded without recurrence, and the parent artery remained patent.Crucially, this represents one of the longest reported follow-ups for an NF1-associated ruptured distal ACA aneurysm, providing definitive evidence of endovascular durability in this rare setting where previous short-term studies lacked recurrence data. At the same time, the follow-up angiogram documented persistent moyamoya syndrome and an untreated aneurysm in the ophthalmic segment of the right internal carotid artery.To our knowledge, this is the first report to simultaneously confirm durable occlusion of an index ruptured aneurysm and document the progressive, multifocal nature of NF1 vasculopathy on long-term angiography—directly validating the mandate for lifelong surveillance even after successful index treatment, a key clinical takeaway missing from most prior case reports (23, 31, 32). This combination suggests that even when the index aneurysm is cured, NF1-related vasculopathy may continue to evolve elsewhere in the cerebrovascular tree. To mitigate the risk of *de novo* aneurysm formation and stroke in such patients, a multimodal prevention and surveillance strategy is critical. For moyamoya-related hemodynamic compromise, revascularization procedures—such as encephaloduroarteriosynangiosis (EDAS) or superficial temporal artery-middle cerebral artery (STA-MCA) bypass—have been shown to improve cerebral perfusion and reduce the stimulus for fragile collateral vessel formation (26, 31). While our patient declined intervention for the new ophthalmic aneurysm, current guidelines recommend proactive management of moyamoya vasculopathy in symptomatic patients or those with high-risk lesions (e.g., aneurysms in collateral-dependent territories). Additionally, regular vascular imaging (CTA/MRA every 1–2 years, with DSA reserved for equivocal findings) is advised to detect *de novo* aneurysms early, given that NF1 patients with moyamoya have a 3–5 fold higher risk of new lesion formation compared to those without moyamoya (23, 32). Therefore, individualized long-term surveillance should be considered, particularly in patients with vascular symptoms, family history, or documented cerebrovascular abnormalities (23, 31, 32). CTA, magnetic resonance angiography, or digital subtraction angiography may all play complementary roles depending on the clinical scenario.

This report has several limitations. It describes only a single patient, and therefore the conclusions cannot be generalized without caution. Detailed molecular testing was not available in the source record, and treatment of the newly identified ophthalmic-segment aneurysm was not performed because the patient declined intervention for financial reasons. Nevertheless, the case provides a useful illustration of treatment decision-making and long-term follow-up in a rare and complex cerebrovascular manifestation of NF1.

## 4 Conclusion

Ruptured A3 segment aneurysm associated with NF1 is extremely rare and may coexist with other forms of NF1-related vasculopathy, including moyamoya syndrome. In the present case, endovascular coil embolization achieved durable exclusion of the ruptured aneurysm with good long-term patency of the parent artery. Because NF1-associated cerebrovascular disease may be multifocal and progressive—and because moyamoya-related vascular fragility increases the risk of *de novo* aneurysm formation in distal and collateral vessels—a comprehensive strategy combining revascularization for moyamoya vasculopathy (when indicated) and lifelong vascular surveillance (CTA/MRA/DSA) is essential to reduce the risk of recurrent hemorrhage or new aneurysm formation, even after successful treatment of the index lesion.

## Data Availability

The raw data supporting the conclusions of this article will be made available by the authors, without undue reservation.
